# Editorial on: Musculoskeletal Rehabilitation: Current Challenges and New Perspectives

**DOI:** 10.3390/jcm12123981

**Published:** 2023-06-12

**Authors:** Andrea Perna, Luca Proietti

**Affiliations:** 1 Departments of Orthopedic and Traumatology, Fondazione Casa Sollievo della Sofferenza, San Giovanni Rotondo, 71013 Foggia, Italy; 2 Department of Orthopaedics and Traumatology, Fondazione Policlinico Universitario A. Gemelli IRCCS—Università Cattolica del Sacro Cuore, 00168 Rome, Italy; luca.proietti@policlinicogemelli.it

Musculoskeletal disorders are among the leading causes of disability and chronic pain worldwide, and their impact is expected to increase in the coming years. Chronic musculoskeletal pain is a disorder that affects millions of people worldwide. It can manifest in various parts of the body, such as the back, knees, or shoulders, and can be caused by various factors including age, intense physical activity, poor posture, and trauma. Chronic pain can limit a person’s ability to perform daily activities and can have a significant impact on quality of life. One of the most common chronic pain conditions is lower back pain, which affects millions of people worldwide. This type of pain is often associated with osteoarthritis of the hips and can lead to a condition known as hip and spine syndrome [1]. Lower back pain can have a significant impact on an individual’s quality of life, often leading to disability, decreased physical activity, and increased healthcare costs. Understanding the underlying causes of lower back pain is critical to developing effective treatments and improving patient outcomes [1,2]. The management of these conditions requires a multidisciplinary approach, involving different healthcare professionals and a wide range of interventions, from pharmacological therapy [2,3] to invasive procedures and non-invasive rehabilitation techniques. In this context, the role of musculoskeletal rehabilitation is crucial, both in the prevention of disability and in the promotion of functional recovery. In recent years, several new techniques have been developed for musculoskeletal rehabilitation, aimed at improving the effectiveness of treatments and reducing their invasiveness. These techniques include innovative physical therapy protocols, such as virtual reality and exergames, as well as regenerative therapies, such as platelet-rich plasma and stem cells. Moreover, new technologies, such as wearable sensors and mobile apps, are being increasingly used for remote monitoring and self-management of musculoskeletal conditions. Despite these advances, several challenges still need to be addressed in musculoskeletal rehabilitation.

In this Special Issue, we aim to discuss the current challenges in musculoskeletal rehabilitation and explore new perspectives on how to overcome them.

One of the main challenges is to ensure that treatments are evidence-based and patient-centered, taking into account the individual needs and preferences of each patient. In addition, it is essential to improve the integration and coordination of care among different healthcare providers, in order to ensure continuity and quality of care throughout the rehabilitation process.

Recent studies have shown the efficacy of non-invasive techniques such as exercise therapy, manual therapy, and modalities such as ultrasound and electrical stimulation in improving musculoskeletal function in conditions such as knee osteoarthritis, rotator cuff tears, and low back pain [4,5,6]. These techniques have been found to be effective both in the pre-operative setting as well as in post-operative rehabilitation.

In addition, the use of minimally invasive techniques such as arthroscopy and percutaneous vertebroplasty has revolutionized the treatment of musculoskeletal conditions, allowing for faster recovery and better outcomes [7,8]. However, there is still debate surrounding the optimal timing and utilization of these techniques in rehabilitation.

Furthermore, the importance of rehabilitation after orthopedic surgery or acute events such as stroke or myocardial infarction cannot be overstated. Studies have shown that early and intensive rehabilitation can lead to improved outcomes in terms of functional status, pain management, and overall quality of life [9,10,11]. Rehabilitation plays a crucial role in the recovery of patients, improving their quality of life and reducing the risk of complications and readmissions. However, the optimal timing, intensity, and duration of rehabilitation interventions are still a matter of debate, and further research is needed to identify the most effective strategies. Another challenge in musculoskeletal rehabilitation is the need for individualized treatment plans. Each patient has unique needs and requires a tailored approach to their rehabilitation. This requires close collaboration between the rehabilitation team, including physical therapists, occupational therapists, and other healthcare professionals, to develop a comprehensive plan that addresses the patient’s specific needs and goals. In addition, patient engagement and education are critical to achieving successful outcomes, as patients must be motivated to actively participate in their own recovery [12,13].

Telemedicine has proven to be an important resource for accessing care, especially for patients with chronic illnesses that require constant medical attention. In particular, rehabilitation through telemedicine has emerged as an innovative solution for the care of patients suffering from chronic musculoskeletal pain. Telemedicine has been adopted as a means to improve access to rehabilitation services for chronic musculoskeletal pain. Tele-rehabilitation allows the patient to be monitored remotely and healthcare services to be delivered effectively and flexibly. Patients can access rehabilitation services more quickly, avoiding long waiting lists in hospitals, and can receive personalized care based on their individual needs [14].

The use of technologies such as videoconferencing, sensors, and smartphones has allowed doctors to provide continuous support to patients in pain management and rehabilitation. Tele-rehabilitation programs include personalized exercises, pain therapies, and relaxation techniques to improve mobility and reduce pain. In addition, tele-rehabilitation also provides psychological support to help patients manage the stress and anxiety associated with chronic pain [15].

Tele-rehabilitation offers numerous advantages over traditional treatments. First, it reduces costs for the patient and the healthcare system as a whole. Furthermore, it offers greater flexibility in scheduling rehabilitation sessions, allowing patients to perform therapies from the comfort of their own home or in any other location they deem appropriate [16]. One promising new perspective in musculoskeletal rehabilitation is the use of regenerative medicine. This approach involves the use of stem cells, growth factors, and other biological materials to promote tissue repair and regeneration [17]. While still in the early stages of development, regenerative medicine has shown promising results in the treatment of musculoskeletal injuries and degenerative conditions such as osteoarthritis. In addition, advances in genetic engineering and personalized medicine may allow for even more targeted and effective regenerative therapies in the future. Finally, it is important to recognize the impact of musculoskeletal conditions on overall health and well-being. Chronic musculoskeletal conditions such as osteoarthritis can lead to decreased mobility, increased pain, and reduced quality of life. As such, a holistic approach to rehabilitation that includes lifestyle modifications, such as diet and exercise, as well as mental health support is critical to improving overall health outcomes for patients with musculoskeletal conditions. 

In conclusion, musculoskeletal rehabilitation is a constantly evolving field that requires innovative approaches and close collaboration between healthcare professionals and patients. Non-invasive techniques, individualized treatment plans, regenerative medicine, and a holistic approach to rehabilitation are all promising new perspectives that can help overcome current challenges and improve patient outcomes. By continuing to explore and develop new approaches, we can ensure that patients receive the best possible care and achieve the highest levels of function and quality of life.
